# Social Network Analysis for Program Implementation

**DOI:** 10.1371/journal.pone.0131712

**Published:** 2015-06-25

**Authors:** Thomas W. Valente, Lawrence A. Palinkas, Sara Czaja, Kar-Hai Chu, C. Hendricks Brown

**Affiliations:** 1 Preventive Medicine, School of Medicine, University of Southern California, Los Angeles, CA, United States of America; 2 School of Social Work, University of Southern California, Los Angeles, CA, United States of America; 3 School of Medicine, University of Miami, Miami, FL, United States of America; 4 School of Medicine, Northwestern University, Chicago, IL, United States of America; University of South Carolina, UNITED STATES

## Abstract

This paper introduces the use of social network analysis theory and tools for implementation research. The social network perspective is useful for understanding, monitoring, influencing, or evaluating the implementation process when programs, policies, practices, or principles are designed and scaled up or adapted to different settings. We briefly describe common barriers to implementation success and relate them to the social networks of implementation stakeholders. We introduce a few simple measures commonly used in social network analysis and discuss how these measures can be used in program implementation. Using the four stage model of program implementation (exploration, adoption, implementation, and sustainment) proposed by Aarons and colleagues [1] and our experience in developing multi-sector partnerships involving community leaders, organizations, practitioners, and researchers, we show how network measures can be used at each stage to monitor, intervene, and improve the implementation process. Examples are provided to illustrate these concepts. We conclude with expected benefits and challenges associated with this approach.

## Social Network Procedures for Program Implementation

Social networks are ubiquitous and arise from interactions between individuals or organizations in many different settings. Social network analysis (SNA) provides a set of theories, techniques, and tools useful for understanding a broad range of human behavior changes as people interact with certain others. For example, one can examine how reproductive health behaviors are transmitted within a village [2] or how HIV is transmitted through a population via sexual partnering [3]. SNA is especially relevant to understanding, aiding, guiding, and improving the program implementation process. In this paper, we concentrate on implementing evidence-based or potentially effective behavior change programs that promote health. The premise of this paper is that attention to the social networks of implementing agencies, change agents, and larger social systems as well as the networks of intervention recipients will substantially improve the implementation process.

This paper briefly introduces the SNA field and then outlines and details ways that SNA can contribute to the implementation process for evidence-based programs, practices, policies, and principles (programs will be used as shorthand for all of these). We begin with a discussion of why SNA matters for program implementation, briefly introduce the core elements of SNA and use social network diagrams for exploratory as well as confirmatory use, characterize an implementation process and the stages of program implementation, and suggest research measures, techniques, procedures, and tools that can be used to apply SNA to implementation. Throughout we draw on examples from our work and that of others. We conclude with a discussion of the potential benefits of this approach to program implementation.

## Why SNA Matters for Implementation

Getting evidence-based programs into practice has increasingly been recognized as a concern in many domains of public health and medicine [4, 5]. Research has shown that there is a considerable lag between an invention or innovation and its routine use in a clinical or applied setting [6]. There are many challenges in scaling up proven programs so that they reach the many people in need [7–9].

There are a number of social processes that are necessary in getting programs adopted, implemented, and sustained. Three that relate directly to social networks and program effects are: (1) partnerships between researchers, community, policy makers, and practitioners that support implementation [10–12], (2) intervention agents (i.e., those who deliver the program), implementation agents, and intermediaries (i.e., those who support the delivery of the program) [13], and (3) the social context of how people receive the program [9]. Many behavior change programs are created or designed in academic or research settings where they are tested under tightly controlled laboratory conditions (i.e. efficacy studies), and then are tested in real-world settings via academic-community partnerships, with the academic partner mostly responsible for maintaining rigorous research methods (i.e. effectiveness studies). Programs that meet high standards for rigorous designs, impact, and replicability in effectiveness trials can earn the label “evidence-based” and be considered by some policy makers and institutions as worthy of consideration for large-scale implementation [14]. This is the traditional translational pipeline flowing from efficacy and effectiveness to implementation research that has driven a large proportion of work in this field. Examples of this research pipeline can be seen in the Blueprints Project, (http://www.colorado.edu/cspv/blueprints/) which has taken highest-level programs and supported their movement into other communities, service delivery agencies, and school systems. Other programs are developed by communities or organizations outside of research settings, and then implemented on large scale without much formal evaluation. Only later does impact on the population become of major evaluation interest. Such programs may continue to be revised in response to effectiveness findings, even as they continue to be implemented at a large scale. Hybrid designs [15, 16], where effectiveness and implementation are examined together in the same study, provide a flexible way to examine and improve the program itself as well as its delivery system, in the context of local as well as global ecological factors and influences.

Partnerships are vital to the successful adoption, implementation and sustainability of successful programs. Indeed, evidence-based programs that have progressed to implementation and translation stages report that effective partnerships with community-based, school, or implementing agencies are critical to their success [11, 17, 18]. Understanding which partnerships can be created and maintained can be accomplished via social network analysis. For example, Valente and colleagues [10] showed that an academic-community intervention to improve cancer screening rates among Asian Pacific Islander communities in southern California was successful at increasing linkage between community based organizations (CBOs) and themselves, and links from CBOs to universities, but not between universities or from universities to CBOs.

A second factor related to effective implementation is the appropriate choice of who (or which organization) delivers the program. Many programs use haphazard, invalid, or convenience methods to identify who delivers the program [19]. Yet interventions implemented by community-identified leaders are often more effective than those by non-leaders [20, 21]. In general, interventions delivered by people from the community of the beneficiaries of the program will be more effective than those delivered by outside agencies that are less connected to program recipients [22, 23].

In an implementation research project led by Chamberlain and colleagues, Palinkas and others [24] collected social network data of key implementation agents. This implementation project compared and tested two alternative implementation strategies for scaling up an evidence-based practice known as Multidimensional Treatment Foster Care (MTFC) in a group of California and Ohio counties. This study found that counties whose service systems leaders were identified by others as a source of information and advice exhibited significantly greater progress in implementing MTFC than counties lacking such leaders. Such networks of policymakers and practitioners are a central feature of many models of dissemination and implementation [1, 25, 26]. This project also showed that a type of learning collaborative, Community Development Teams, designed to strengthen the network between counties as a way of resolving implementation challenges, (1) strengthened the network [27], (2) led to no improvement in speed or stage of implementation [15], and (3) improved the quality and quantity of implementation [15].

A third factor is the social context in which the program is received. Studies have shown that networks can mediate intervention effects. That is, intervention effects may vary as a function of the recipients’ social networks [28, 29]. For example, Shin and others [29] demonstrated that children with friends who were physically inactive gained more from an obesity prevention program than those with physically active friends. Wyman and others [30] hypothesized that a peer-led suicide prevention program would have differential effects based on how peripheral or isolated a youth was. In sum, network theories and techniques can help understand factors related to successful program implementation.

## What Is SNT/SNA?

Social network theory and analysis (SNTA) is a field of research that has emerged over the past 100 years from a niche discipline to applications spanning many fields of the social, physical, and biological sciences. There are core tenets and principles as well as widely used software, visualization, and analytic tools. In the applications presented in this paper, we focus primarily on social networks of individuals during development, design, implementation, and monitoring of behavior change programs, but acknowledge these networks could also be of organizations, agencies, coalitions, and so on. We review four measures used in this description but the interested reader can consult other resources to learn of the many other measures commonly used in social network research [21, 31–33].

Social network analysis is conducted by recording data on who is connected to whom. These relations can be many and varied; and can be derived from survey information (e.g. who is friends with whom) or archival traces such as email exchanges, joint purchasing behavior, joint authorship behavior, or GPS co-location information, to name but a few. The network data are used to derive individual network measures such as the number of links each individual has, and network level measures such as network density, a count of the number of links present expressed as a proportion of all links possible.

There are numerous network measures available and the measures introduced here are meant to be illustrative, not exhaustive. In this regard, a few simple definitions will suffice. Components are the subgroups in networks. A component is the set of all people in the network that are reachable via any number of steps. A network can have one or multiple components. Density is the number of links in the network expressed as a proportion of all links possible. Individual centrality measures the extent a person occupies a prominent or important position in the network; people on the periphery are many steps away from the center. Isolates are people with no links in the network, yet they are within the network boundary. Centralization assesses the extent links are focused around one or a few people. Reciprocity occurs when a relationship in one direction also occurs in the opposite direction: Bob and Mary report knowing one another. Transitivity occurs when two people who are connected to one another are also connected to a third person: Friends of friends are friends.

**Fig 1:**
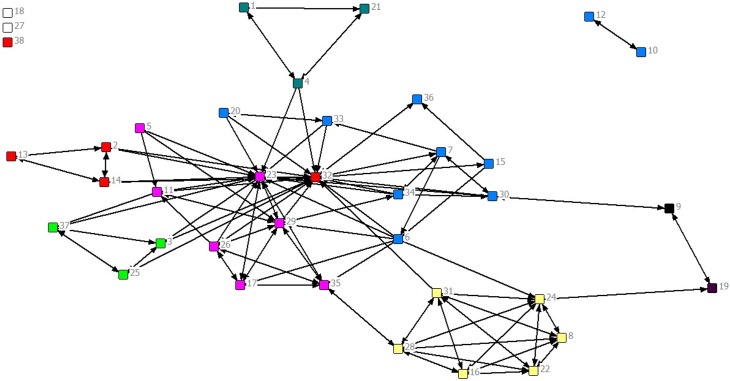
Sample organizational network with nodes colored by department.

We will use the network depicted in Fig 1 to illustrate some network principles. These data represent the extent to which 37 members of an organization know each other. Each circle is a person and the number inside the circle his/her ID number. Lines connecting people depict who knew whom with an arrow signifying a direction in that relationship. For example, 19 and 24 report knowing each other, but 15 reported knowing 36 yet 36 did not report knowing 15.

## Frameworks for the Stages of Implementation

In order to apply social network theory and methods to implementation, we note first that implementation can be considered a developmental process, a transition through known stages [1, 34]. Here, we use a four stage model of implementation progress [1], which is similar to those used in evaluation frameworks [35] and in the diffusion of innovations [25, 36]. The four stages are: (1) Exploration or needs assessment; (2) Adoption or program design; (3) Program implementation, and (4) Sustainment and monitoring.

The first stage, exploration, involves a broad assessment of the community to gain a deep understanding of the community’s needs, vision, and opportunities for change. While this phase is carried out somewhat differently by different prevention systems, there are some common elements in having communities and organizations decide what program would meet their needs, how adaptations may be needed, and how an effective delivery system can be built within the home organization. At some level, all action steps involve developing a comprehensive mapping of the community boundaries and population to be served, and the organizations and political constituencies that are present. A first use of social networks in implementation involves the exploration of these constituents’ mutual self-interests and relationships to one another as this provides the attractive forces to establish, grow, and sustain a coordinated coalition. Such information can be used to assess the social capital [37] available that can be mobilized as a resource to support program implementation and sustainment. Expressing and maintaining this self-interest is necessary as coalition members and their constituents often have different or competing interests as well. Information about each individual stakeholder’s interests, their vision for their community, and their connections to other constituencies can be assembled from face-to-face interviews (including recommendations of whom they think the coalition should talk to next), their public relations with others, and digital footprints, particularly a summary of email and phone contacts. Indeed, this mapping has also been described by Kellam [23] as a critical first stage before a community undertakes the implementation of an evidence-based program.

During this initial assessment, the development team should (1) understand barriers and facilitators to the behavior(s) of interest, (2) identify additional community partners, leaders, and gatekeepers who can help develop and/or implement the program, (3) identify ecological, and delivery system issues that may affect program implementation, and (4) gather any data needed to benchmark both the behaviors (such as the rate of obesity or substance use), and community and organizational factors that can affect implementation (e.g., demographics, resources, culture, and context).

The second stage involves the adoption of an existing program or the creation of an intervention to address the behavior problem. There are many evidence-based programs available for use in prevention and treatment of physical, mental and behavioral health problems (see for example: http://www.cdc.gov/hiv/prevention/programs/ebis/). Most programs, however, need to be adapted to local settings, which is considered in the exploration phase and developed during the adoption phase. If a program is adopted, the implementing agents and/or agency may need to adapt the existing fidelity monitoring tool designed for that program to accurately track the delivery and implementation of the adapted program. Social network data may be important in the adoption stage so as to understand who makes the decision on which interventions to adopt and whether and how they need to be adapted. For instance, social networks have been viewed as an important characteristic of community coalitions [38–41]. Feinberg and colleagues [42] found network cohesion to be positively associated and network centralization to be negatively associated with community readiness to engage in the Communities That Care community-based prevention coalition. Bess and colleagues [38] found that initial coalition participation in a youth violence prevention program was associated with a pre-existing network of inter-organizational relations.

The third stage is program implementation with fidelity, which involves delivering the program with adherence and competence in real-world settings [43]. Program implementation research focuses on how the program is delivered and research is designed to determine if variation in program outcomes are associated with variations in program execution. Increasingly, researchers are learning that program effects demonstrated under idealized or laboratory conditions often do not translate into sustainable programs in practice [44, 45]. A key area where networks can be of use in this phase is examining the process of monitoring fidelity of the intervention agent(s), reviewing by a supervisor, and providing feedback in a timely fashion. A dynamic network, measuring the timing and degree of information exchange between a single intervention agent and a supervisor, as well as between intervention agents and the intended beneficiaries of the program (e.g., attendance at meetings, homework completion), could be used to measure qualities expected to predict improved outcomes.

The final stage is sustainment and monitoring which occurs as we attempt to determine if the program continues to be implemented as intended over time and is continuing to have the anticipated effects. Monitoring long-term outcomes of programs is often neglected under the assumption that if the program works, then it will continue to work and benefit the communities receiving the program. The reality is that programs change and drift, the relevance of programs change, populations and communities served by these programs change, and the people implementing the programs change [46]. Any and all of these changes can affect a program’s delivery, reach, fidelity, and impacts among intended audiences. Current theories suggest that sustainability is affected not only by which communities in a network are represented in a supportive network, but also how early this network begins, whether there is a “champion” and/or whether the network represents the power structures in organizations who have the capacity to address long term financial and administrative viability [47, 48]. Network analyses tools provide ways to monitor programs so their continued sustainability can be assessed.

## Network Measures and Metrics Useful for Program Implementation

### Exploration and Needs Assessment

There are at least five actions interventionists can perform using social network techniques during the exploration /needs assessment phase: (1) determine if there is a network, (2) identify isolated or marginal individuals or groups, (3) identify individuals or groups to engage in program design, (4) determine if there are subgroups that might need to be brought together, and (5) determine if individual or group attributes are associated with network ties or structures. First, network measures can be used to determine if there is a network. In some settings, there may be no formal or informal network in place to implement an intervention program. In such cases, it may be important to form a network. For example, many underserved communities do not have existing neighborhood councils, community groups, or informal networking opportunities that can provide support and/or advice on health topics and to help establish the importance of the program. Sometimes forming a network is easy and public health specialists or community activists merely need to find a place and time to meet. Other times, however, this scientific model of implementing evidence-based programs may be at odds with the communities’ values, experiences, and resources. It is critical to establish a balanced partnership from the beginning [23], in order to avoid an imbalance in power favoring an academic over a community perspective which would then make it difficult to sustain programs over time [49].

Thus a developmental perspective on the network is important as is the topology and the link to organizations’ power structure. In these cases, sometimes forming a network relies on a charismatic and highly motivated leader, and when this happens, it should be documented as program replication may require identifying such a person. There is tension here, however. If programs rely on this charismatic leader to get established and maintained, the program is at risk because it depends on this person’s commitment, availability, and resources. To successfully transition to later stages of implementation, the program will require a deeper commitment by other community members, leaders, and stakeholders. Often the program will need to become institutionalized somehow.

Therefore it is important to document how the original network was formed and what strategies and tactics were employed to develop and grow the network. Critical to successful network formation is the “Goldilocks Principle” that it has just the right amount of density; centralization/decentralization; core-peripheriness; variation among stakeholders; and high cohesiveness/low fragmentation. Determining the right levels of density, centralization, clustering and so on can be difficult and will be context specific.

Since these metrics vary from zero to one, a helpful guide would be to consider levels below 0.30 as low and, depending on the context, amenable to be increased. Levels in the 0.30 to 0.50 range are moderate and might be considered appropriate. Levels above 0.50 are probably too high in many situations and may impede diffusion, performance, or collective action, among other outcomes. Even this suggestion must be tentative however since even the most basic measure, network density, has been posited to be advantageous for spreading new information but deleterious when accepting new information from the outside [11]. And in one intervention, increased density was found to inhibit adoption of evidence-based programs [50]. In addition, there is rarely just one network that evolves; rather, there are often multiple networks, at multiple layers, with multiple perspectives that need to be coordinated.

The second function of network analysis at this stage is to determine if there are individuals, groups, or organizations that are isolated or marginally connected to existing networks. In Fig 1, nodes 18 and 27 are isolated from the network, and this isolation makes it difficult for them to receive information, resources, or services that may be necessary to maintain their health or healthy lifestyles. This may be an area for the implementation of strategies that foster expanded network linkages. Note also in Fig 1, persons 10 and 12 are connected to each other but disconnected from the main group, which means they also may be in a disadvantaged network position; persons 9 and 19 have few connections to the group and may be outside the normal flow of information.

The third function of network analysis in the needs assessment phase is to use the network information to identify individuals or groups that can and should be solicited to help identify community needs, barriers to change, and positive motivations for change. Typically, this would entail identifying individuals who are central in the network and thus can be thought of as representative of the community’s needs. Care must be exercised however to insure that the network analysis has not simply identified the power brokers in a community but indeed has identified those that everyone thinks is important. Although there are many measures that identify central nodes in a network [33, 51], only some are appropriate for use in the exploration phase. We wish to identify nodes that are prominent in the network, whom many people consider important or whom they trust and/or would go to for advice. At the same time, we want these individuals to be broadly representative of the community so they span the network most efficiently. In other words, we want to avoid identifying a set of individuals that are interconnected and represent the same subgroup in the network. Thirdly, we would like to identify individuals whose first-hand experiences with the program are positive or are positively inclined towards using evidence-based programs (e.g., scoring high on an instrument such as the EBPAS [52] or demonstrating skills required for implementation). Borgatti [53] developed the Key Player program, which provides precisely the measure we seek, identifying important nodes that represent different subgroups in the network. Key Player is a tool that researchers can use to identify important nodes, while ensuring that these nodes are located in different sub-sections of the network so the voices from various factions are heard and incorporated into the needs assessment. Combining EBPAS with network measures would be a logical set of tools for this exploratory phase.

**Fig 2:**
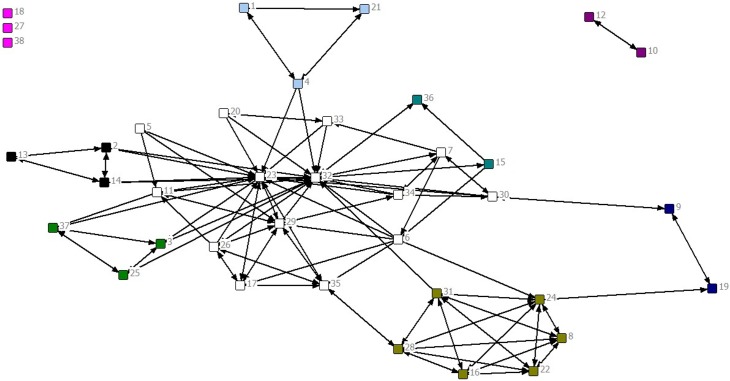
Network with nodes colored by their groups based on a community detection algorithm.

The fourth function of network analysis within the exploratory phase is to determine if there are subgroups in the network that should become inter-connected or that need to be addressed separately. There are many community detection algorithms developed to determine the extent to which a network can be described as consisting of subgroups [54, 55]. The algorithms provide a measure of modularity, called Q, which indicates how much the network is clustered within sub-groups or communities identified by the analysis. Q values greater than 0.30 indicate a reasonably strong group structure and those above 0.50 indicate a very strong group structure. The network in Fig 1 has a Q value of 0.40 and the analysis identified 8 groups in addition to the two isolates. Fig 2 colors the nodes in the network by their groups.

Finally, it is important to determine if individual attributes are associated with network connections and structure. In Fig 1, the nodes are colored by the departments within the organization for each person. It is apparent that many of the nodes connect solely or primarily to others in their department. The yellow cluster is almost entirely contained within itself, and there is a tendency for the grays, reds, and blues to be inter-connected. (Indeed, in a statistical model, network members in the same department were more likely to be connected to one another.) The influence of individual attributes is important to consider since the attributes may be strongly associated with the behavior under investigation and they may provide clues for how to stimulate change in the network. For the network in Fig 1, it is clear that links across departments may need to be created to allow more rapid flow of information and influence in the network. In many cases, individual sex, ethnicity, education, age, religious affiliation, or economic status may influence network linkages in different ways and may create barriers to effective network functioning.

As in all network research, deciding exactly which networks to measure and how to measure them can be challenging. For the needs assessment phase, the network analysis should identify individuals “who can motivate the community to be healthy.” In other words, ask network questions that will identify individuals who would be best suited to helping with identifying community problems and solutions. In many cases, a full census of the community may not be possible and so a snowball approach in which key informants are recruited and they name others that should be consulted [56]. Some sample of the people named in the first wave should then be interviewed, typically those that receive some threshold level of nominations; and potentially some named in the second wave. Many studies follow protocols that recruit individuals from different positions of leadership such as criminal justice, education, government, media, healthcare services and so on [57]. Unfortunately, many studies fail to document these procedures and so research is needed on how community leaders and partners are recruited to participate in health promotion interventions.

### Program design

Once satisfied that a successful needs assessment has been conducted, the implementation team can turn to the task of designing the intervention either by adapting an existing evidence-based one so that it meets the needs of the community, or designing a new program. Research has shown that who delivers the program, and the social context of its receipt has a strong impact on program appeal and impact [22, 58]. Program recipients do not interpret program materials in a vacuum; rather they interpret the content within their social and environmental contexts either explicitly or implicitly. Social network analysis can be used at this stage in at least five ways: (1) identifying opinion leaders to act as change agents, (2) using community members as recruiting agents, (3) consideration of other network interventions methods, (4) consideration of the social context of program delivery, and (5) attending to social media and other communication needs.

One of the most frequently used network intervention tactics has been the recruitment and training of peer identified opinion leaders to implement behavior change programs [19, 20, 22]. Network-identified peer opinion leaders have been used in dozens of studies to accelerate behavior change. Most of these studies have been conducted among physicians or other service providers in health care settings. These studies have relied on using network in-degree, a count of the number of times a person is named in response to a network question, to identify the leaders or champions for change. Opinion leader interventions are effective because they solicit community input to determine who should implement the program, and because the leaders come from the community they often have considerable trust and social capital. Moreover, since the leaders know the community, they can help suggest necessary program adaptations during implementation. Finally, because the leaders come from the community and are embedded within the community, the interventions have the potential to be more sustainable [59]. However, despite their leadership role within the community, it is important to ensure that the opinion leaders are well trained in the intervention.

Many interventions have used community members as recruitment agents. There has been a long history of successful HIV prevention interventions using peer networks to reach individuals at risk such as injection drug users (IDUs) [60]. The most common intervention has been the respondent driven sampling (RDS) approach in which IDUs who have received a behavioral intervention are then given coupons to distribute within their social networks so these peers can then also receive the intervention [61]. The initial indexes and their alters are both incentivized to participate. These RDS interventions have been shown to be quite effective at reducing risk behavior [62].

Many other network interventions are possible, and Valente [22] classified them into four broad strategies, each comprised of several tactical alternatives with each tactic having from one to many operational choices. For example, opinion leader interventions fall under the strategy of selecting individual change agents, but tactically for some programs one might choose to identify bridges, or marginal, or low threshold adopters. Operationally, if one selects leaders, there are dozens of specific mathematical algorithms that identify the most central nodes. Other strategies include network segmentation, induction, and alteration. Space prohibits review of all of these and interested reader is directed to consult [22]. Selecting a network intervention from this wide array of strategic, tactical and operational choices depends on the behavior of study, the goals and objectives of the intervention, the characteristics of the population, and the context in which the intervention will be implemented.

The fourth use of network analysis for program design is consideration of the social context of program receipt. Many interventions are designed to be delivered in group settings in part to increase their cost-effectiveness and in part to harness the power of group dynamics to facilitate behavior change [63]. Alcoholics and Narcotics Anonymous interventions use group meetings to encourage commitment to behavior change; and even media campaigns would be wise to consider the social context of message receipt [64]. In short, designing interventions that encourage social networks to reinforce behavior change messages are likely to be more effective than those that ignore it.

Finally, the increasing importance of social media use among all populations means that public health interventions are likely to re-imagined and re-interpreted with a social media lens [65]. Program participants will tweet and post their reactions to intervention materials and activities. If positive, these actions may increase program participation and effects and can in some cases take on a life of their own. Designers are thus encouraged to consider how their interventions may be adapted to online platforms, how to create content amenable to social media, and how to encourage participants to use social media to increase program effects.

In the program design stage researchers usually measure networks of expertise and trust operationalized as “who do you go to for advice” and “who do you discuss problems with”[66, 67]. The advice question identifies individuals who are knowledgeable about the issue at hand and have the necessary technical know-how to persuade non-users. The discussion question identifies individuals who are trustworthy and who are “people people” and good communicators. When the barriers to change are technical, advice networks are important; when the barriers to change are cultural, then discussion ones are important. In other words, when people report they do not know how to do the behavior, advice or expertise is needed, but when people report they do not know what behaviors to do, discussion or trust is needed. There is strong evidence that opinion leader and RDS interventions are effective behavior change approaches [20, 68]. There is some evidence that identifying leaders and forming groups around them [58]; or forming groups and identifying leaders within them are also effective approaches [69]. What is lacking is comparison between network intervention methods. For example, do opinion leader approaches work better than snowball (RDS) ones; and to what extent does the kind of network measured influence network intervention effectiveness (i.e., are advice networks more effective than discussion ones).

### Implementation

Most interventions are not one shot “single-time, single dose,” but instead are implemented over many months and involve many sessions. For example, school-based interventions typically occur over 10–12 weeks with a single lesson each week during one class period. Similarly, many community-based interventions are delivered in group settings with multiple sessions, often facilitated by a leader or interventionist who in some cases may also vary. There are many network tools and statistics that can be used to facilitate program implementation and enable mid-course program adjustments.

Gesell and colleagues [70] introduced the concept of network diagnostics in which network data are collected and analyzed during program implementation in order to provide diagnostic information useful to the program’s implementation during its delivery. These investigators worked with a 12-week program designed to improve lifestyle choices for parents in underserved communities. The intervention taught healthy shopping, cooking, eating, and lifestyle choices in a group format. The theory guiding the intervention was that increased social cohesion during the group sessions would improve information seeking and social support among group members, which would translate into better outcomes. At two intervals during the intervention (weeks 3 and 6), network data were collected and the results of the network analysis were given to the interventionist and provided specific advice on activities to use to increase group cohesiveness.

This advice was based on the following metrics [70]: (1) isolates—they should specifically be included in activities, (2) asymmetry in connections—this should be minimized, (3) density—which should be greater than 15% but less than 50%, (4) subgroups—there should be no distinct subgroups, everyone should be able to reach everyone else in the network either directly or indirectly, (5) centralization—this should be minimized so no one individual or group dominates the network, and (6) transitivity and cohesion—these group characteristics should be encouraged but limited. The recommendations regarding appropriate metrics and thresholds for these metrics were based primarily on experiences with one pilot study, and much work remains to be done in this area. However, this example demonstrates the usefulness of the network analysis approach in the implementation stage of an intervention.

During the implementation stage, researchers should measure the same networks as the ones measured in the design stage. The most salient measures will be to determine the centrality of those who have changed their behavior in response to the intervention. In addition, it is important to identify any marginal individuals or groups who may not be responding to the intervention and who thus need to be recruited into participating more fully. A further consideration at this stage is to make an assessment of whether the intervention is changing the networks: Are intervention agents becoming more or less central in the network. We know of only one study, Gesell and others [70], that has used network data during the implementation stage so clearly more experience is needed to determine whether networks can be used to assist program implementation.

### Sustainment and Monitoring

After the program has been implemented, the hope of most interventionists is that the program will continue to be implemented and the impact of the program on outcomes sustained over the long term. Many studies have deplored the tendency for programs to lose funding, focus, or purpose once the initial investments are removed [71, 72]. And unfortunately many effective programs are discontinued prematurely and many ineffective ones continued when they should be terminated [73]. We expect that following the guides provided here using network analysis to aid in needs assessment, program design, and implementation will result in programs that are more sustainable than ones that do not consider the importance of social networks. Here, we specify procedures for using network data to document program sustainability, and suggest remedies should the evidence indicate a lack of sustainability.

The dynamic systems framework proposed by Chambers and others [74] provides a model that is consistent with the network proscriptions provided here, namely that continuation of a program provides added opportunity for learning and adaptation of the program rather than attempting to freeze it in place. There are at least four metrics/network analytic techniques that can be used to aid in the evaluation of sustainability: (1) documentation of the continuation of the network; (2) documentation of the behavior of leaders; (3) estimation of contagion (or learning) effects; and (4) strategies to help ensure that participants hear positive things about the program from their peers.

As in the program design phase, one task in the sustainability phase is to be sure there is a network relevant to the program. Asking participants whether they continue to discuss the content of the program and whether lessons learned are incorporated into the day-to-day interactions of the community is important to document as evidence that the program is still relevant. This type of process data should be routinely included in sustainability evaluations.

Aside from assessing whether the community in general is aware of and values the program, it is also important to document that the program is still relevant to important community leaders and gatekeepers. Again, it is important to know whether central individuals embrace the program and still see it as relevant and a priority. This type of data collection and monitoring should be included in standard program evaluations so that researchers know the network positions of their informants. Interventionists may often think the program is still being embraced by the community because the people they come into contact with regularly still report that it is important. But these individuals may not be central in the network for which the programs are developed and so the surveillance information available to the interventionist is not a valid reflection of what is happening in the community.

Most programs generate their effects either directly or indirectly through interpersonal persuasion and communication [75]. Individuals change their behaviors because they perceive themselves to have the personal supports to change, because they see the new behavior as normative, and because they see how it benefits themselves and those around them [21]. Sustainable programs are ones for which these perceptions are maintained among program participants. Network analyses can document these perceived normative influences and determine if participants maintain a perception that their interpersonal environment supports and endorses the new behaviors. Moreover, truly successful programs turn program participants into advocates, and network analyses can demonstrate whether participants provide positive influences to their peers. For example, with a standard network survey researchers can ask whether participants discussed program components with one another.

Finally, related to the last point above, sustainable programs have created “buzz” that makes people excited about the program and its success, especially within various social media platforms. Researchers need to document what people think about the program and what they hear “on the street” about it. While not specific to SNA, they can also measure current perceptions of the program and determine if recent participants’ experience of the program is similar to earlier participants. The important consideration is to assess how people discuss the program, and if they do, whom they discuss it with. Often, we need to answer the question: Are program graduates recruiting new people?

Chambers and others [74] write (p. 118) “As a consequence, assessment of organizational characteristics (e.g., structure, climate, culture, resources) is seen as an essential component of sustainability, and indeed, the fit between context and the intervention is at the center of a sustainability phase.” A central tenet of this network paper is that a key organizational characteristic to assess is the network context of program delivery and receipt. By monitoring the network of delivery agents and organizations, researchers can more adequately determine how the program is changing and how its effects may vary.

During the sustainment and monitoring stage the same networks measured for program design should be assessed. Researchers should also ask program recipients whether they have had contact with intervention change agents. For analyses, researchers should test whether there is behavioral contagion within the community and among program recipients to others in the community [76]. Researchers also need to determine if prominent/central network members embrace the behavior being promoted. This is also an opportunity to monitor social media platforms to determine what people say about the intervention and whether it is becoming part of the community discourse. There are many studies showing that networks are important influences on behavior change, but few that directly assess whether intervention agents influence program recipients.

Table 1 summarizes the research questions, common network measures used, concepts, and outcomes when applying network analysis methods to these program implementation stages.

**Table 1:** Network Analyses Procedures for each Stage of Implementation.

	Stage of Implementation			
	Exploration (Needs Assessment)	Adoption (Program Design)	Implementation	Sustainment & Monitoring
Research Questions	Who is recruited to design the intervention?	Are community leaders/ opinion leaders engaged as change agents?	Are network structure metrics at appropriate levels?	Do central individuals and/or organizations remain involved and committed?
	Who defines the needs?	Who delivers the intervention and what is the social network of its receipt?	What is the network position of early adopters/users?	Does the network exhibit changes conducive to continued program success?
Measures	Density Isolates/MarginalsKey Players Groups By Attributes	Strategies: Individuals; Segmentation; Induction; and Alteration	Density Isolates Symmetry Groups Centralization Transitivity/Cohesion	Density Leaders/Central Nodes Contagion Advocacy
Concept	Network Ethnography	Network Interventions	Network Diagnostics	Network Surveillance
Outcomes	Document network position and structure of those providing input into problem definition.	Select network properties of intervention design.	Use network data to inform and modify intervention delivery.	Ensure continued program use by important network nodes.
Citation Example		Valente, 2012 [22]	Gesell et al., 2013 [70]	Iyengar et al., 2010 [75]

## Potential Benefits

Using network data to inform all stages of program development and implementation offers considerable potential benefits in a number of areas. First, it will substantially advance theory about how programs work and how best to design and implement them. Human service interventions are designed, delivered, and consumed by people; and for most people, the most important aspect of their lives are their social networks. Interventions can be conceived as programs that enable people to build and maintain better, health-promoting, and supportive networks in addition to individual changes in cognitions, attitudes, and/or behaviors.

Existing evidence indicates that network-informed interventions and programs are more effective than non-networked ones. All programs think about networks and most probably acknowledge that networks are important components of program design and delivery. Yet, most programs do not explicitly account for networks in their design, delivery, and evaluation. Network data will enable us to improve our programs without substantially increasing costs or revising existing interventions. Indeed, it is likely that network-based interventions are more cost-effective because of their stronger impacts and because local buy-in and delivery are enhanced.

Collecting network data has become easier in today’s digital world. Mining and analyzing online networks makes use of accurate data, is cost-effective, and may be less obtrusive than pen-and-paper surveys. Online data can be used at any point during the different stages of program implementation, whether it is from emails for coordination, or fully integrating social media websites into an intervention. Much of the technology that supports online interactions is inherently designed to connect different persons and groups, and thus is ideal for network exploration. As more methods of communication become digital, researchers need to capitalize on the plethora of network data that can be made available. In addition, experience indicates that collecting network data via surveys, either electronically or with paper, is easy and efficient. Everyone knows who they go to for advice or whom they spend time with.

Finally, incorporating network data into program design and intervention may enable us to conceive of new interventions. For example, school-based programs are often created with the intention of delivering the same content to all students. Yet, we know that students cluster into identifiable cliques and groups. Consequently, it makes sense to design separate interventions for different groups or cliques of students, particularly when those groups are distinct. For example, a school-based group of “jocks” may benefit from different programs than a group of “nerds.”

## Conclusions

This brief introduction to the application of social network analysis for program implementation is not comprehensive and highlights that much work remains to be done. Many of the suggestions provided here are based on limited evidence at this point in time, but the available evidence is promising. Social network analyses can be applied to intervention development and implementation to foster and enhance the implementation process. There is no rigorous comprehensive database of network experiences for this applied work at the present time. There is, however, a wealth of knowledge about how to collect and analyze social network data, and a plethora of measures, both individual- and network-level, that can be used to improve the design, delivery, and assessment of public health interventions.
